# Relative muscle mass and the risk of incident type 2 diabetes: A cohort study

**DOI:** 10.1371/journal.pone.0188650

**Published:** 2017-11-30

**Authors:** Sungwoo Hong, Yoosoo Chang, Hyun-Suk Jung, Kyung Eun Yun, Hocheol Shin, Seungho Ryu

**Affiliations:** 1 Center for Cohort Studies, Total Healthcare Center, Kangbuk Samsung Hospital, Sungkyunkwan University School of Medicine, Seoul, Korea; 2 Department of Occupational and Environmental Medicine, Kangbuk Samsung Hospital, Sungkyunkwan University School of Medicine, Seoul, Korea; 3 Department of Clinical Research Design & Evaluation, SAIHST, Sungkyunkwan University, Seoul, Korea; 4 Department of Family Medicine, Kangbuk Samsung Hospital, Sungkyunkwan University School of Medicine, Seoul, Korea; The University of Tokyo, JAPAN

## Abstract

**Aims:**

The association between relative muscle mass (RMM) and incidence of type 2 diabetes (T2DM) is largely unknown. We examined whether RMM predicted development of T2DM in an apparently young healthy population.

**Methods:**

This cohort study was comprised of 113,913 men and 89,854 women, free of T2DM at baseline, who underwent a health checkup examination and were followed-up annually or biennially for an average of 2.9 years. We used skeletal muscle mass index (SMI) as an indicator of RMM. SMI (%) [total skeletal muscle mass (kg)/body weight (kg)×100] was estimated using a bioelectrical impedance analyzer. The study outcome was incident T2DM, defined as fasting serum glucose ≥126 mg/dL, HbA1C ≥6.5%, or use of medication for T2DM.

**Results:**

During 589,098.8 person-years of follow-up, 4,264 individuals developed T2DM (incidence rate, 7.2 per 1000 person-years). Median age (range) at baseline was 39.1 years (18.1–87.1). RMM was negatively associated with incidence of T2DM in a dose-response manner. The multivariate-adjusted hazard ratios (95% CIs) for incident T2DM comparing quartiles 3, 2 and 1 of RMM to the highest quartile were 1.32 (1.14–1.52), 1.63 (1.42–1.86), and 2.21 (1.94–2.51), respectively, for males and 1.18 (0.88–1.58), 1.46 (1.11–1.91), and 1.96 (01.51–2.53) for females (P for trend <0.001; 0.011). This association was stronger in younger or premenopausal subjects.

**Conclusions:**

RMM was negatively associated with development of T2DM in a large sample of young and middle-aged Korean adults. Further research is required to determine whether preservation of muscle mass through intervention affects the risk of T2DM.

## Introduction

The prevalence of type 2 diabetes (T2DM) is rapidly increasing worldwide [1]. T2DM is associated with considerable morbidity and mortality, which contributes to the global health burden [2]; however, the causes of T2DM are not fully understood. Thus, it is important to identify modifiable risk factors to establish preventive strategies for this disease.

Sarcopenia is age-related loss of skeletal muscle mass and function [3,4], and is associated with adverse health outcomes such as frailty, T2DM and mortality [5,6]. Recently, studies have shown that relative muscle mass (RMM) is closely related to metabolic and cardiovascular risk factors [7,8]. These associations have been reported not only in the elderly population, but also in the young and middle-aged populations, which supports the importance of muscle mass in a population with a wide range of ages [9].

Skeletal muscle comprises a large proportion of body mass and plays a key role in glucose metabolism via glucose consumption and storage [10,11]. Several cross-sectional studies of the elderly population have suggested an association between sarcopenia and T2DM patients [12,13]. Furthermore, a cross-sectional study of a middle-aged population (mean age: 41.0 years) demonstrated that RMM was inversely associated with insulin resistance and prediabetes; this finding was observed across the full ranges of RMM, and was not limited to the sarcopenic range [7]. Thus, we hypothesized that RMM could affect the development of T2DM over time, even in the non-elderly population. Recently, a prospective study of older adults reported no overall association between muscle mass and risk of incident T2DM, although the results were inconsistent and varied by subgroups [14].

Therefore, we examined the association between RMM and the development of T2DM in a large cohort of young and middle-aged Korean men and women that did not have T2DM at baseline and that participated in a health screening examination program.

## Materials and methods

### Study population

The Kangbuk Samsung Health Study is a cohort study of Korean men and women that underwent a comprehensive annual or biennial health examination at the Kangbuk Samsung Hospital Health Screening Centers in Seoul and Suwon, South Korea [15,16]. Over 80% of participants were employees of various companies and local governmental organizations, and their spouses. In Korea, annual or biennial health screening exams of employees are required by the Industrial Safety and Health Law examinations. The remaining participants voluntarily attended the health checkup program.

**Fig 1:**
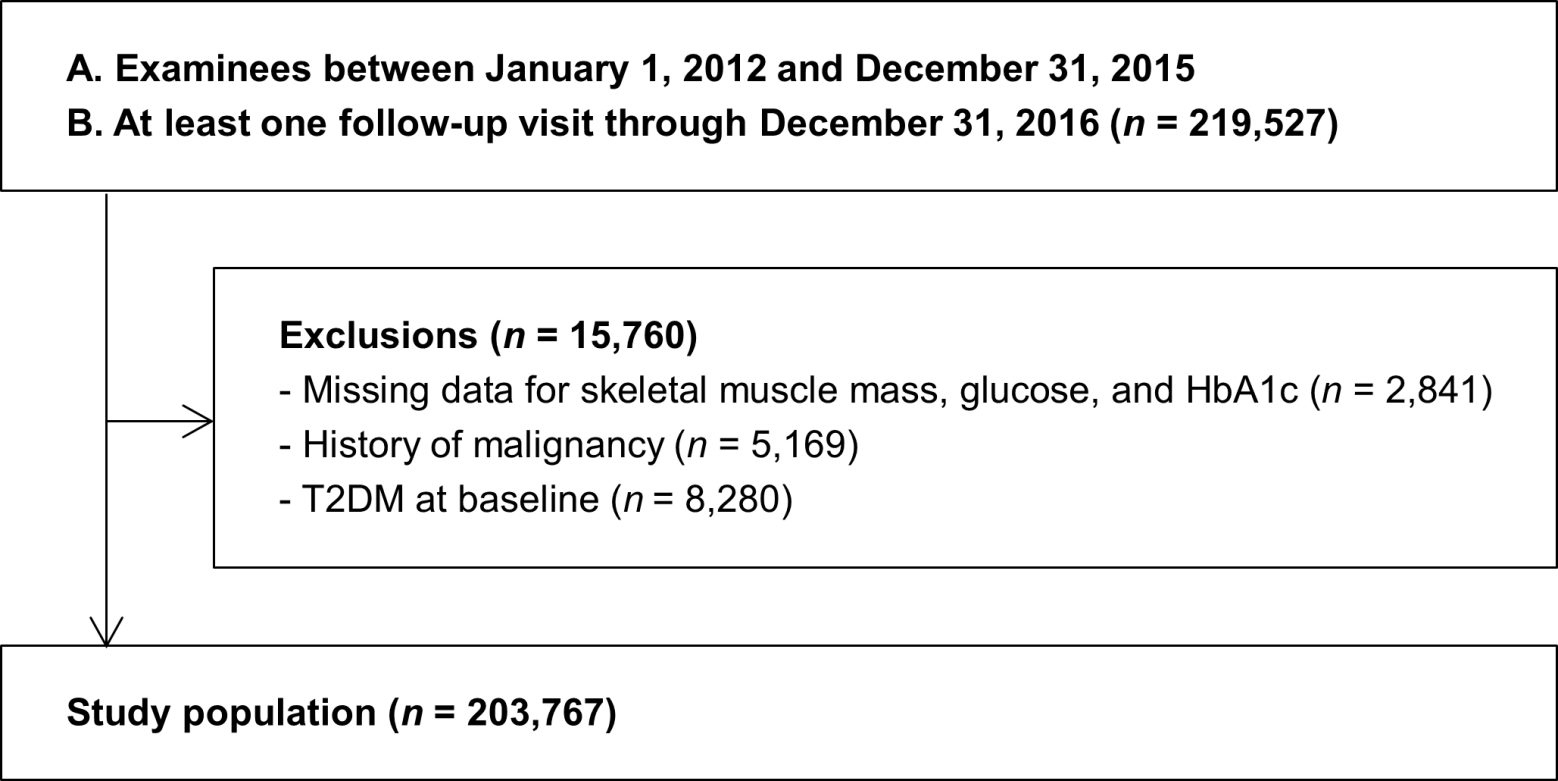
Selection of study population. Of the people who were examined between January 1, 2012 and December 31, 2014, 219,527 people had at least one follow-up visit by December 31, 2016, and 203,767 were eventually included in the study. T2DM, type 2 diabetes mellitus.

The study population consisted of examinees who underwent a comprehensive examination between January 1, 2012 and December 31, 2014 and who had at least one follow-up visit through December 31, 2016 (*n* = 219,527). We excluded participants who had any of the following conditions at baseline: missing data for skeletal muscle mass, glucose and HbA1c (*n* = 2,841), history of malignancy (*n* = 5,169), or T2DM at baseline (*n* = 8,280). Because some individuals met more than one exclusion criterion, the total number of subjects included in the study was 203,767 (Fig 1).

The study was approved by the Institutional Review Board of the Kangbuk Samsung Hospital, which waived the requirement for informed consent as we used only de-identified data obtained as part of routine health screening exams.

### Measurements

Data on demographic characteristics, smoking status, alcohol consumption, education level, medical history, and medication use were also collected by standardized, self-administered questionnaires, as previously described [15,17,18].

We measured physical activity level and sitting time using the validated Korean version of the international Physical Activity Questionnaire (IPAQ) Short Form [19,20]. Physical activity levels were categorized as previously described [18]. Health-enhancing physically active (HEPA) was defined as physical activity that meets either of two criteria: (i) vigorous intensity activity on three or more days per week accumulating ≥1500 MET min/week; or (ii) seven days of any combination of walking, moderate intensity, or vigorous intensity activities achieving at least 3000 MET min/week [21].

Typical dietary consumption was assessed using a 106-item self-administered food frequency questionnaire (FFQ) designed and validated for use in Korea [22]. The validity and reproducibility of our FFQ were evaluated previously by comparing nutrient and food intake derived from twelve 24-hour dietary recalls during four seasons and with a second FFQ administered one year later. A database of recipes, portion size, and nutrients was constructed with a food composition table from the Korean Nutrition Society.

Percentage of body fat and skeletal muscle mass were estimated using a multi-frequency bioimpedance analyzer with eight-point tactile electrodes (InBody 720, Biospace Co., Seoul Korea), which was validated with respect to reproducibility and accuracy for body composition [23]. Height, weight, and body composition were measured by trained nurses with the participants wearing a lightweight hospital gown and no shoes. BMI was calculated as weight in kilograms divided by height in meters squared and obesity was defined the proposed cutoff for the diagnosis of obesity in Asians [24].

Skeletal muscle mass index (SMI) was calculated as SMI (%) = skeletal muscle mass [kg]/body weight [kg] ×100, based on the methods by Janssen et al. [25].

Serum biochemical parameters were measured, including glucose, uric acid, hemoglobin A1c, insulin, total cholesterol, triglycerides, low-density lipoprotein cholesterol (LDL-C), and high-density lipoprotein cholesterol (HDL-C) and are described in detail elsewhere [17]. Insulin resistance was assessed with the homeostatic model assessment–insulin resistance (HOMA-IR) equation: fasting blood insulin (uU/ml) × fasting blood glucose (mmol/l) / 22.5. The Laboratory Medicine Department at Kangbuk Samsung Hospital in Seoul, Korea is accredited by the Korean Society of Laboratory Medicine (KSLM) and the Korean Association of Quality Assurance for Clinical Laboratories (KAQACL), the laboratory participates in CAP (College of American Pathologists) Survey Proficiency Testing. Blood pressure was measured using an automated oscillometric device (Welch Allyn 5300P) while subjects were in a sitting position with the arm supported at heart level. Hypertension was defined as a systolic blood pressure ≥ 140 mmHg, diastolic blood pressure ≥ 90 mmHg, or current use of antihypertensive medication. Prediabetes was defined as a fasting glucose 100~125 mg/dL or HbA1c 5.8~6.4%. T2DM was defined as a fasting serum glucose ≥ 126 mg/dL, hemoglobin A1c ≥ 6.5%, or current use of insulin or anti-diabetic medications. Menopause was defined as the absence of your menstrual cycle for one year (12 months) and was collected via questionnaire.

### Statistical analyses

Characteristics of the study participants were explored according to RMM index. RMM was categorized into the following gender-specific quartiles [7]: for men 26.6–41.6%, 41.7–43.6%, 43.7–45.5%, and 45.6–71.2; for women, 14.3–36.0%, 36.1–38.2%, 38.3–40.4%, and 40.5–62.6%. Category numbers were used as continuous variables in regression models to test for linear trends.

Development of T2DM was designated as the primary endpoint. Each participant was followed from the baseline exam until development of T2DM, with the assumption of a date of diagnosis in the midpoint between the assessment at which T2DM was diagnosed and the previous assessment or the last health exam conducted prior to December 31, 2016, whichever came first. The incidence rates were calculated as the number of incident cases divided by person-years of follow-up. A Cox proportional hazards model was used to estimate the adjusted hazard ratios with 95% confidence intervals (CI) for incident T2DM. Since T2DM was known to have developed between the two visits but the precise time at which it developed was unknown, a parametric Cox model was used to account for this type of interval censoring (*stpm* command in Stata) [26]. In these models, the baseline hazard function was parameterized with restricted cubic splines in log time with four degrees of freedom. Because men and women had different body compositions, multivariate analysis was conducted in men and women separately. Three models with progressively increased adjustment were used for confounding variables. The basic model was adjusted for age, and then, additional adjustments were then made for study center (Seoul, Suwon), year of screening exam, smoking (never, past, current, or unknown), alcohol intake (0, <20g/d, ≥ 20g/d, or unknown), physical activity (no HEPA *vs*. HEPA), educational level (high school graduate or less, community college or university graduate, graduate school or higher and unknown), total calorie intake (quintiles or unknown), history of cardiovascular disease, history of hypertension, medication for dyslipidemia, family history of T2DM, and menopause (only for women). Finally, the analysis was further adjusted for HOMA-IR, fasting glucose and hsCRP. We assessed the proportional hazards assumption by examining graphs of estimated log (-log) survival. Number of categories was used as a continuous variable and tested on each model to determine linear trends of incidence.

In addition, we performed stratified analyses in pre-specified subgroups defined by age (<50 *vs*. ≥50 years), smoking (current smoker *vs*. noncurrent smoker), physical activity (no HEPA *vs*. HEPA), BMI (<25 kg/m^2^ *vs*. ≥ 25 kg/m^2^), HOMA-IR (<2.5 *vs*. ≥ 2.5), hsCRP (<1.0 mg/l *vs*. ≥ 1.0 mg/l), prediabetes status (normoglycemia *vs*. prediabetes) and menopause (No *vs*. Yes, in female). The interactions by subgroup characteristics were tested using likelihood ratio tests, comparing models with and without multiplicative interaction terms.

All P-values were two-tailed, and values of *P* < 0.05 were considered statistically significant. We used STATA version 14.0 (Stata Corp., College Station, TX, USA) for data analysis.

## Results

Baseline characteristics of study participants and categories of RMM are presented in Table 1 and Table 2 by sex. The mean (SD) age and BMI of men at baseline were 39.5 (7.5) years and 24.4 (2.9) kg/m^2^, respectively. The mean (SD) age and BMI of women at baseline were 38.5 (7.5) years and 21.6 (2.9) kg/m^2^, respectively. Men and women in lower RMM categories were more likely to be older, obese, and drink alcohol; more likely to have history of cardiovascular diseases, hypertension, and use of dyslipidemia medication; have family history of T2DM; had higher levels of body fat percentage, fasting glucose, systolic blood pressure, diastolic blood pressure, total cholesterol, LDL-C, triglycerides, hepatic enzymes, HOMA-IR, and hsCRP; and had lower levels of HDL-C and total energy intake (Tables 1 and 2). In addition, women in lower RMM categories were more likely to have experienced menopause.

**Table 1:** Baseline characteristics: Relative muscle mass of male participants.

Characteristics	Overall	Relative muscle mass category^a^					*P* for trend
		Q1 (26.6–41.6%)	Q2 (41.7–43.6%)	Q3 (43.7–45.5%)		Q4 (45.6–71.2%)	
**Number**	113,913	28,481	28,480		28,475	28,477	<0.001
**Age (years)**^**b**^	39.5 (7.5)	39.9 (7.8)	40.1 (7.5)		39.7 (7.3)	38.4 (7.1)	<0.001
**BMI (kg/m**^**2**^**)**	24.4 (2.9)	27.1 (2.8)	24.8 (2.1)		23.7 (2.0)	22.1 (2.1)	<0.001
**Percent fat mass (%)**	22.6 (5.3)	29.4 (3.1)	24.1 (1.2)		20.9 (1.2)	16.1 (2.6)	<0.001
**Obesity (%)**	38.6	77.1	44.9		24.5	7.9	<0.001
**Current smoker (%)**	35.7	36.5	35.0		35.5	36.0	0.383
**Alcohol intake (%)**^**c**^	34.0	35.4	34.7		34.5	31.2	<0.001
**HEPA (%)**	16.1	12.1	14.1		16.6	21.6	<0.001
**Highest education level (%)**^**d**^	88.6	89.0	89.1		88.9	87.5	<0.001
**History of CVD (%)**	1.0	1.2	1.1		1.0	0.8	<0.001
**History of hypertension (%)**	9.7	15.7	10.0		8.0	5.2	<0.001
**Family history of diabetes (%)**	14.5	15.5	15.0		14.6	13.2	<0.001
**Medication for dyslipidemia (%)**	2.8	4.3	3.1		2.4	1.5	<0.001
**Prediabetes (%)**	37.6	46.8	39.9		35.3	28.3	<0.001
**Systolic BP (mmHg)**^**b**^	112.6 (11.2)	116.1 (11.8)	113.1 (11.0)		111.7 (10.5)	109.6 (10.3)	<0.001
**Diastolic BP (mmHg)**^**b**^	73.6 (9.3)	76.3 (9.8)	74.2 (9.2)		72.9 (8.9)	70.8 (8.6)	<0.001
**Glucose (mg/dl)**^**b**^	95.3 (8.2)	96.8 (8.6)	95.9 (8.2)		95.0 (8.0)	93.5 (7.8)	<0.001
**Total cholesterol (mg/dl)**^**b**^	199.9 (33.9)	208.2 (34.8)	203.1 (33.7)		198.7 (32.8)	189.7 (31.2)	<0.001
**LDL-C (mg/dl)**^**b**^	127.6 (30.8)	135.5 (31.2)	131.0 (30.5)		126.9 (29.8)	117.2 (28.8)	<0.001
**HDL-C (mg/dl)**^**b**^	52.8 (12.7)	49.1 (11.1)	50.9 (11.6)		53.1 (12.4)	57.9 (13.6)	<0.001
**Triglycerides (mg/dl)**^**e**^	113 (80–162)	138 (101–193)	123 (89–173)		109 (80–154)	87 (64–119)	<0.001
**ALT (U/l)**^**e**^	23 (17–33)	30 (21–45)	24 (18–35)		21 (16–30)	18 (14–24)	<0.001
**AST (U/l)**^**e**^	21 (18–26)	24 (19–30)	22 (18–27)		21 (17–25)	20 (17–24)	<0.001
**GGT (U/l)**^**e**^	29 (20–47)	40 (27–63)	32 (22–51)		27 (20–42)	22 (16–31)	<0.001
**HOMA-IR**^**e**^	1.35(0.90–1.98)	1.88 (1.31–2.67)	1.47 (1.03–2.06)		1.25 (0.85–1.76)	0.97 (0.65–1.39)	<0.001
**hsCRP (mg/l)**^**e**^	0.5 (0.3–1.0)	0.8 (0.5–1.5)	0.6 (0.3–1.0)		0.5 (0.3–0.8)	0.3 (0.2–0.6)	<0.001
**Total energy intake (kcal/d)**^**e**^^,^^**f**^	1633.9 (1308.3–2016.6)	1627.9 (1293.9–2020.5)	1621.4 (1299.8–1994.0)		1628.3 (1311.6–2001.6)	1660.3 (1327.2–2048.4)	<0.001

ALT, alanine aminotransferase; AST, aspartate aminotransferase; BMI, body mass index; BP, blood pressure; CVD, cardiovascular disease; GGT, gamma-glutamyl transferase; HDL-C, high-density lipoprotein-cholesterol; HEPA, health-enhancing physical activity; HOMA-IR, homeostasis model assessment of insulin resistance; hsCRP, high sensitivity C-reactive protein.

^a^Skeletal muscle index quartile level. Men: quartile 1, 26.6–41.6%; quartile 2, 41.7–43.6%; quartile 3, 43.7–45.5%; quartile 4, 45.6–71.2%.

Data are ^b^means (standard deviation)

^e^medians (interquartile range), or percentages.

^c^≥ 20 g of ethanol per day

^d^≥College graduate.

^f^Among 79,250 participants with plausible estimated energy intake levels (within three standard deviations from the log-transformed mean energy intake).

**Table 2:** Baseline characteristics: Relative muscle mass of female participants.

Characteristics	Overall	Relative muscle mass category^a^				*P* for trend
		Q1 (14.3–36.0%)	Q2 (36.1–38.2%)	Q3 (38.3–40.4%)	Q4 (40.5–62.6%)	
**Number**	89,854	22,465	22,462	22,466	22,461	<0.001
**Age (years)**^**b**^	38.5 (7.5)	40.1 (8.9)	38.8 (7.5)	38.0 (6.8)	37.3 (6.1)	<0.001
**BMI (kg/m**^**2**^**)**	21.6 (2.9)	24.5 (3.2)	21.9 (2.0)	20.6 (1.7)	19.4 (1.6)	<0.001
**Percent fat mass (%)**	29.0 (5.8)	36.4 (3.3)	30.8 (1.5)	26.9 (1.4)	21.9 (2.6)	<0.001
**Obesity (%)**	11.5	37.4	7.2	1.3	0.2	<0.001
**Current smoker (%)**	2.0	2.1	1.8	1.8	2.3	0.065
**Alcohol intake (%)**^**c**^	5.5	6.2	5.2	5.4	5.1	<0.001
**HEPA (%)**	13.4	11.3	12.5	13.4	16.4	<0.001
**Highest education level (%)**^**d**^	77.9	71.5	78.0	80.6	81.7	<0.001
**History of CVD (%)**	0.5	0.8	0.7	0.4	0.4	<0.001
**History of hypertension (%)**	3.0	6.0	2.9	1.9	1.3	<0.001
**Family history of diabetes (%)**	17.4	19.3	18.0	16.9	15.3	<0.001
**Medication for dyslipidemia (%)**	1.4	2.6	1.4	1.0	0.5	<0.001
**Prediabetes (%)**	28.2	36.3	28.4	25.1	23.1	<0.001
**Menopause (%)**	8.2	13.7	8.8	6.1	4.3	<0.001
**Systolic BP (mmHg)**^**b**^	99.5 (10.5)	103.0 (11.9)	99.4 (10.2)	98.1 (9.6)	97.3 (9.2)	<0.001
**Diastolic BP (mmHg)**^**b**^	64.2 (8.3)	66.1 (9.0)	64.2 (8.3)	63.5 (8.0)	63.1 (7.8)	<0.001
**Glucose (mg/dl)**^**b**^	90.6 (7.7)	92.5 (8.3)	90.8 (7.6)	89.9 (7.4)	89.1 (7.1)	<0.001
**Total cholesterol (mg/dl)**^**b**^	108.9 (28.7)	120.2 (30.9)	111.2 (28.1)	105.0 (26.3)	99.3 (24.6)	<0.001
**LDL-C (mg/dl)**^**b**^	108.9 (28.7)	120.2 (30.9)	111.2 (28.1)	105.0 (26.3)	99.3 (24.6)	<0.001
**HDL-C (mg/dl)**^**b**^	64.9 (14.5)	60.5 (14.0)	63.8 (14.2)	66.2 (14.2)	69.1 (14.2)	<0.001
**Triglycerides (mg/dl)**^**e**^	69 (54–94)	84 (63–116)	72 (55–97)	66 (52–87)	61 (48–77)	<0.001
**ALT (U/l)**^**e**^	13 (10–16)	14 (11–19)	12 (10–16)	12 (10–15)	12 (10–15)	<0.001
**AST (U/l)**^**e**^	17 (15–20)	17 (15–21)	17 (15–20)	17 (15–19)	17 (15–20)	<0.001
**GGT (U/l)**^**e**^	13 (10–17)	15 (11–21)	13 (10–17)	12 (10–16)	12 (10–16)	<0.001
**HOMA-IR**^**e**^	1.07 (0.72–1.54)	1.41 (0.95–2.05)	1.12 (0.76–1.58)	0.99 (0.68–1.38)	0.87 (0.60–1.22)	<0.001
**hsCRP (mg/l)**^**e**^	0.3 (0.2–0.6)	0.6 (0.3–1.3)	0.3 (0.2–0.7)	0.3 (0.2–0.5)	0.2 (0.2–0.4)	<0.001
**Total energy intake (kcal/d)**^**e**^^,^^**f**^	1404.4 (1061.8–1772.0)	1410.1 (1066.9–1787.0)	1397.3 (1057.2–1762.8)	1399.8 (1058.0–1760.0)	1410.7 (1064.4–1776.4)	0.787

ALT, alanine aminotransferase; AST, aspartate aminotransferase; BMI, body mass index; BP, blood pressure; GGT, gamma-glutamyl transferase; HDL-C, high-density lipoprotein-cholesterol; HEPA, health-enhancing physical activity; HOMA-IR, homeostasis model assessment of insulin resistance; hsCRP, high sensitivity C-reactive protein.

^a^Skeletal muscle index quartile level. Women: quartile 1, 14.3–36.0%; quartile 2, 36.1–38.2%; quartile 3, 38.3–40.4%; quartile 4, 40.5–62.6%.

Data are ^b^means (standard deviation)

^e^medians (interquartile range), or percentages.

^c^≥ 20 g of ethanol per day

^d^≥College graduate.

^f^Among 62,820 participants with plausible estimated energy intake levels (within three standard deviations from the log-transformed mean energy intake).

During 589,098.8 person-years of follow-up, 4,264 subjects developed T2DM (incidence rate, 7.2 per 1000 person-years). The average follow-up period was 2.9 years. The association between RMM category and T2DM development was examined in men and women separately (Table 3). The RMM category was negatively associated with an increased incidence of T2DM in a dose-response manner. The interaction of sex and RMM category in T2DM development was statistically significant (*P* for interaction < 0.001). Low RMM was more strongly associated with risk of T2DM in men compared to women. In age-adjusted models, the hazard ratios (95% CIs) for T2DM when comparing quartiles 1–3 *vs*. quartile 4 of RMM were 3.80 (3.38–4.26), 2.31 (2.04–2.60), and 1.69 (1.49–1.92), respectively, in men. After adjusting for study center, year of screening exam, smoking, alcohol intake, physical activity, educational level, total caloric intake, family history of T2DM, history of cardiovascular disease, history of hypertension, and medication for dyslipidemia, the hazard ratios (95% Cis) for incident T2DM when comparing quartiles 1–3 of RMM to the highest quartile were 3.56 (3.17–4.01), 2.25 (1.99–2.55), and 1.65 (1.45–1.88), respectively, in men (Table 3, Model 1) (*P* for trend < 0.001). In age-adjusted models, the hazard ratios (95% CIs) for T2DM when comparing quartiles 1–3 *vs*. quartile 4 of RMM were 4.50 (3.58–5.65), 2.32 (1.82–2.97), and 1.50 (1.15–1.96), respectively, in women. The hazard ratios (95% Cis) for incident T2DM when comparing quartiles 1–3 of RMM to the highest quartile were 3.26 (2.58–4.11), 1.98 (1.55–2.54), and 1.42 (1.09–1.85), respectively, in women (*P* for trend < 0.001) (Table 3, Model 1). We performed additional analyses adjusted for HOMA-IR, fasting glucose and hsCRP to determine if the increased risk of T2DM, associated with RMM category, was mediated by insulin resistance and low-grade inflammation. Additional adjustment for HOMA-IR, fasting glucose and hsCRP slightly reduced the associations, but they remained modest and statistically significant in both sexes (Table 3, Model 2).

**Table 3:** Development of type 2 diabetes by relative muscle mass.

Relative muscle mass category	Person-years	Incident case	Incidence Density (per 1,000 person-years)	Age-adjusted HR (95% CI)	Multivariate HR^a^(95% CI)	
					Model 1	Model 2
**Men**						
** Q1 (26.6–41.6%)**	78,761.2	1,382	17.5	3.80 (3.38–4.26)	3.56 (3.17–4.01)	2.21 (1.94–2.51)
** Q2 (41.7–43.6%)**	82,935.2	896	10.8	2.31 (2.04–2.60)	2.25 (1.99–2.55)	1.63 (1.42–1.86)
** Q3 (43.7–45.5%)**	84,944.9	654	7.7	1.69 (1.49–1.92)	1.65 (1.45–1.88)	1.32 (1.14–1.52)
** Q4 (45.6–71.2%)**	86,768.8	370	4.3	1.00 (reference)	1.00 (reference)	1.00 (reference)
***P* for trend**				<0.001	<0.001	<0.001
** Per 1 SD decrease in relative muscle mass**				1.67 (1.62–1.73)	1.63 (1.58–1.69)	1.38 (1.32–1.43)
**Women**						
** Q1 (14.3–36.0%)**	60,422.3	496	8.2	4.50 (3.58–5.65)	3.26 (2.58–4.11)	1.96 (1.51–2.53)
** Q2 (36.1–38.2%)**	63,811.3	234	3.7	2.32 (1.82–2.97)	1.98 (1.55–2.54)	1.46 (1.11–1.91)
** Q3 (38.3–40.4%)**	64,918.8	142	2.2	1.50 (1.15–1.96)	1.42 (1.09–1.85)	1.18 (0.88–1.58)
** Q4 (40.5–62.6%)**	66,536.3	90	1.4	1.00 (reference)	1.00 (reference)	1.00 (reference)
***P* for trend**				<0.001	<0.001	0.011
** Per 1 SD decrease in relative muscle mass**				1.37 (1.34–1.40)	1.38 (1.34–1.42)	1.25 (1.19–1.32)

BMI, body mass index; CI, confidence intervals; HR, hazard ratios.

*P* < 0.001 for the overall interaction between sex and RMM categories for development of type 2 diabetes (model 1).

^a^Estimated from parametric Cox models. Multivariate model 1 was adjusted for age, center, year of screening exam, smoking status, alcohol intake, physical activity, education level, total caloric intake, history of cardiovascular disease, history of hypertension, medication for dyslipidemia, family history of diabetes and menopause (only for women): Model 2 was adjusted for the variables in model 1 plus adjustment for HOMA-IR, fasting glucose and hsCRP.

1 SD of relative muscle mass: 3.04 for men and 3.18 for women.

In prespecified subgroup analyses, the inverse association between RMM category and incident T2DM was consistently observed across various clinically relevant subgroups (Table 4); The association between RMM category and incident T2DM differed by age group, obesity, and menopausal status (P for interaction<0.001). The association was stronger in younger individuals (age <50 years) (vs. age ≥50 years; P for interaction < 0.001), and in premenopausal women (vs. postmenopausal women; P for interaction = 0.001).

**Table 4:** Hazard ratios^a^ (95% CI) of incident diabetes according to relative muscle mass category in clinically relevant subgroups.

Subgroup	Relative muscle mass category^b^				*P* for trend	*P* for interaction
	Q1	Q2	Q3	Q4		
**Age**						<0.001
**<50 years (*n* = 160,169)**	4.78 (4.26–5.36)	2.69 (2.38–3.04)	1.76 (1.54–2.00)	reference	<0.001	
**≥50 years (*n* = 20,428)**	2.10 (1.64–2.69)	1.37 (1.06–1.78)	1.34 (1.02–1.75)	reference	<0.001	
**Smoking**						0.468
**Never (*n* = 123,799)**	3.87 (3.37–4.45)	2.20 (1.90–2.54)	1.55 (1.33–1.81)	reference	<0.001	
**Current smoker (*n* = 36,105)**	4.58 (3.80–5.53)	2.59 (2.12–3.16)	1.90 (1.55–2.34)	reference	<0.001	
**HEPA**						0.122
**No (*n* = 149,167)**	4.08 (3.62–4.60)	2.44 (2.15–2.77)	1.66 (1.45–1.90)	reference	<0.001	
**Yes (*n* = 27,768)**	3.82 (3.08–4.72)	1.89 (1.50–2.38)	1.65 (1.31–2.08)	reference	<0.001	
**BMI**						<0.001
**<25 kg/m**^**2**^ **(*n* = 131,471)**	1.75 (1.49–2.07)	1.75 (1.52–2.01)	1.49 (1.30–1.70)	reference	<0.001	
**≥25 kg/m**^**2**^ **(*n* = 49,125)**	1.80 (1.44–2.26)	1.30 (1.03–1.64)	1.08 (0.84–1.38)	reference	<0.001	
**HOMA-IR**						0.141
**<2.5 (*n* = 160,783)**	2.44 (2.16–2.76)	1.87 (1.66–2.12)	1.52 (1.34–1.72)	reference	<0.001	
**≥2.5 (*n* = 18,854)**	2.18 (1.64–2.90)	1.54 (1.15–2.07)	1.13 (0.82–1.56)	reference	<0.001	
**hsCRP**						0.141
**<1.0 mg/l (*n* = 115,824)**	3.01 (2.62–3.46)	2.03 (1.76–2.33)	1.51 (1.30–1.74)	reference	<0.001	
**≥1.0 mg/l (*n* = 33,974)**	4.02 (3.17–5.09)	2.39 (1.86–3.07)	1.51 (1.15–1.98)	reference	<0.001	
**Prediabetes**						0.587
**No (*n* = 113,858)**	2.59 (1.88–3.56)	1.64 (1.17–2.28)	1.14 (0.80–1.62)	reference	<0.001	
**Yes (*n* = 66,194)**	2.89 (2.59–3.23)	1.91 (1.70–2.15)	1.49 (1.32–1.69)	reference	<0.001	
**Menopause (in women)**						0.001
**No (*n* = 69,029)**	4.70 (3.67–6.03)	2.27 (1.73–2.96)	1.38 (1.03–1.85)	reference	<0.001	
**Yes (*n* = 8,573)**	2.97 (1.59–5.56)	1.74 (0.90–3.38)	1.84 (0.93–3.67)	reference	<0.001	

BMI, body mass index; HEPA, health-enhancing physical activity; HOMA-IR, homeostasis model assessment of insulin resistance; hsCRP, high sensitivity C-reactive protein.

^a^Estimated from parametric Cox models adjusted for age, center, year of screening exam, smoking status, alcohol intake, regular exercise, education level, total caloric intake, history of cardiovascular disease, history of hypertension, medication for dyslipidemia, family history of diabetes and menopause.

^b^Skeletal muscle index quartile level. Men: quartile 1, 26.6–41.6%; quartile 2, 41.7–43.6%; quartile 3, 43.7–45.5%; quartile 4, 45.6–71.2%. Women: quartile 1, 14.3–36.0%; quartile 2, 36.1–38.2%; quartile 3, 38.3–40.4%; quartile 4, 40.5–62.6%.

The relationship of RMM and incident T2DM was weakened after further adjustment for BMI (S1 Table); however, the association remained significant. The hazard ratio (95% CIs) for comparing the RMM lowest quartile to the highest quartile was 1.24 (1.10–1.40). The relationship of percent fat mass (%) and incident T2DM was also analyzed after further adjustment for BMI (S2 Table). The hazard ratio (95% CIs) for comparing the percent fat mass (%) lowest quartile to the highest quartile was 1.44 (1.27–1.64).

## Discussion

In this large cohort study of young and middle-aged Korean adults, RMM was inversely associated with a risk of developing T2DM. This association persisted even after adjusting for possible confounders and metabolic parameters and was consistently observed across clinically relevant subgroups. This inverse relationship was observed in a dose-response manner across quartiles of muscle mass. Our findings suggest a protective effect of muscle mass against developing T2DM.

A cross-sectional study by Srikanthan et al. showed that skeletal muscle mass is inversely associated with insulin resistance and the prevalence of prediabetes, in a representative sample of middle-aged adults [7], limiting their ability with temporal ambiguity. A prospective cohort study by Larsen et al. investigated the association between lean body mass and strength with incident T2DM in an elderly population, aged 70 to 79 years [14]. In that study there was no overall association between lean body mass, muscle area, muscle strength and incident T2DM in older adults, limiting the generalizability to the non-elderly population. Another cohort study by Li et al. examined the relationship of muscle mass determined by DEXA with incident diabetes in middle aged men (mean age of 54.1) and reported no association between muscle mass and diabetes [27]. In comparison, we found an inverse association between RMM and the development of T2DM over time in apparently healthy, relatively younger men and women with a mean age of 38.5. Interestingly, the association between RMM and T2DM was much stronger in younger (<50 years) than in older participants; thus the difference in age composition across studies could partly explain why previous studies failed to identify an association between muscle mass and incident T2DM. Our result derived from a much younger population might have been less affected by comorbidities or medication use compared to that in older population.

The mechanisms for relatively low muscle mass and how it can contribute to the development of T2DM have not been fully elucidated. Potential mechanisms that link low muscle mass to incident T2DM can be mediated by insulin resistance, a key pathogenic mechanism of T2DM [7,28]. Systemic inflammation can also be a predictor for developing T2DM [29]. In our study, the association between RMM and incident T2DM was evident even after adjusting for HOMA-IR, fasting glucose and hsCRP. After further adjustment for BMI, the association between RMM and risk of T2DM was attenuated but remained significant, suggesting the independent role of muscle mass on the development of T2DM that is not fully explained by insulin resistance, systemic inflammation, or adiposity.

Skeletal muscle is one of the largest organ groups in the body, plays an important role in glucose metabolism, and is the main site of insulin-mediated glucose uptake [30]. In humans, skeletal muscle takes up 80–90% of glucose in the blood during hyperinsulinemia-euglycemia status [31]. Lower skeletal muscle mass can reduce the capacity to uptake glucose from the blood. Moreover, in recent decades, skeletal muscle has been identified as a secretory organ that produces some cytokines or peptides, which are expressed and released by muscle fibers and designated as myokines including interleukin-6 (IL-6), IL-8, IL-13, IL-15, irisin and myostatin [32]. Some myokines might be involved in the pathogenesis and development of T2DM [33]. Additionally, there is a growing body of evidence showing that skeletal muscle-pancreas crosstalk regulates insulin secretion, possibly mediated by myokines [32]. Unfortunately, this study was not able to include data on these cytokines or peptides, and future studies that assess information about these cytokines or peptides will be required to evaluate this possibility.

In this study, the association between RMM and the risk of incident T2DM was stronger in the younger age group and premenopausal group even though the absolute incidence was higher in the older age group and postmenopausal group. In our study, stronger associations in younger or premenopausal individuals were less likely to be affected by survival bias or bias induced by comorbidities or medication use, which might be present in older individuals. Indeed, skeletal muscle mass starts to decline in the 30s and then declines rapidly in the 50s [34]. Preventive strategies for preserving muscle mass from early to middle age could be helpful to reduce the risk of T2DM, leading to improved health outcome. Future research is required to determine whether preservation of muscle mass through appropriate intervention affects the risk of T2DM and when the intervention should be started. In addition to muscle mass, factors related to muscle quality including muscle strength and composition can affect muscle function [35,36]. Age-related changes in muscle mass and composition can lead to insulin resistance with a reduced capacity for insulin-mediated glucose disposal [36]. In our study, other factors including muscle strength were not available. Recently, the negative impact of grip strength on the risk of T2DM was demonstrated [27]. Like that research, further studies with measures of muscle mass as well as quality such as muscle architecture(aerobic capacity, intramuscular adipose tissue, and muscle fibrosis) will be helpful to determine the role of muscle in the pathogenesis of T2DM [37]. Due to the use of multiple comparisons, chance might be another possible explanation for the observed difference across subgroups.

This study had some limitations. First, information on muscle strength was not available; however, muscle mass has been significantly correlated with muscle strength [38]. Second, we used the BIA method but not DEXA, a gold standard of body composition, to measure muscle mass. The BIA method has been validated against DEXA and is acceptably reliable for body composition [39]. Third, percent fat mass was not adjusted for in the multivariable model. This is due to its strong correlation with RMM as a main exposure in this study (r = 0.97). It is not possible to adequately separate the contributions of these variables to the development of T2DM because of the inherently collinear relationship between percent fat mass (%) and RMM [40]. Fourth, T2DM was diagnosed based on single measurements of fasting glucose and HbA1c without repeated measurement confirmation; however, HbA1c has good pre-analytical stability and is less likely to be affected by acute perturbations (e.g., stress, exercise, or smoking) [41,42]. Impaired glucose tolerance was not measured in this study since the 2-h glucose test was not feasible for a large population-based study. About 2.6% of adults aged ≥20 years with fasting glucose in the non-diabetic range have undiagnosed diabetes based on the 2-h glucose test [43]; thus, the small size of potential misclassification may not affect overall findings. Fourth, a relatively short follow-up period is a limitation of this study. Given that decreased muscle mass has been reported to lower insulin sensitivity even in the short term, our study findings support the important effect of RMM on the development of T2DM [44]. Further studies with longer follow-up are required to determine the long-term effect of muscle mass on the development of T2DM. Finally, the subjects included in this study consisted of young and middle-aged Koreans who regularly attended health-screening exams; thus, our findings might not generalize to other age groups, to populations with a higher prevalence of comorbidities, or to other race / ethnicity groups.

In young and middle-aged Korean populations, RMM was inversely associated with a risk of T2DM in a dose-response manner. RMM may play an independent role in the pathogenesis of T2DM and can be considered as a modifiable risk factor for this disease. Further research is required to determine whether preservation of muscle mass through intervention affects the risk of T2DM.

## Supporting information

S1 Table Development of incident diabetes by relative muscle mass category with adjustment for BMI. (DOCX)

S2 Table Development of incident diabetes by percent fat mass(%) category with adjustment for BMI. (DOCX)
