# Supplementary material for: Relative muscle mass and the risk of incident type 2 diabetes: A cohort study
Source: PLoS One. 2017 Nov 30;12(11):e0188650. doi: 10.1371/journal.pone.0188650 (PMC5708784; doi:10.1371/journal.pone.0188650)
Supplement: S1 Table — (DOCX) [file pone.0188650.s001.docx]

**S1 Table. Development of incident diabetes by relative muscle mass category with adjustment for BMI.**

|  | **Skeletal muscle index category ^a^** | | | | ***P* for trend** |
| --- | --- | --- | --- | --- | --- |
|  | **Q1** | **Q2** | **Q3** | **Q4** |  |
| **Multivariate HR^b^ (95% CI)** | 1.24 (1.10-1.40) | 1.26 (1.13-1.42) | 1.21 (1.08-1.37) | reference | <0.001 |

BMI, body mass index; CI, confidence interval.

^a^Skeletal muscle index quartile levels. Men: quartile 1, 27.6-41.6%; quartile 2, 41.7-43.6%; quartile 3, 43.7-45.5%; quartile 4, 45.6-71.2%. Women: quartile 1, 14.3-36.0%; quartile 2, 36.1-38.2%; quartile 3, 38.3-40.4%; quartile 4, 40.5-62.6%.

^b^Estimated from parametric Cox models. Multivariate model was adjusted for age, center, year of screening exam, smoking status, alcohol intake, physical activity, education level, total calorie intake, family history of diabetes and BMI at baseline.
