# Supplementary material for: Relative muscle mass and the risk of incident type 2 diabetes: A cohort study
Source: PLoS One. 2017 Nov 30;12(11):e0188650. doi: 10.1371/journal.pone.0188650 (PMC5708784; doi:10.1371/journal.pone.0188650)
Supplement: S2 Table — (DOCX) [file pone.0188650.s002.docx]

**S2 Table. Development of incident diabetes by percent fat mass(%) category with adjustment for BMI.**

|  | **Percent fat mass(%) category ^a^** | | | | ***P* for trend** |
| --- | --- | --- | --- | --- | --- |
|  | **Q1** | **Q2** | **Q3** | **Q4** |  |
| **Multivariate HR^b^ (95% CI)** | 1.44 (1.27-1.64) | 1.32 (1.17-1.49) | 1.27 (1.13-1.44) | reference | <0.001 |

BMI, body mass index; CI, confidence interval.

^a^Percent fat mass(%) quartile levels. Men: quartile 1, 2.9-19.1%; quartile 2, 19.2-22.5%; quartile 3, 22.6-26.0%; quartile 4, 26.1-50.2%. Women: quartile 1, 2.9-24.9%; quartile 2, 25.0-28.8%; quartile 3, 28.9-32.9%; quartile 4, 33.0-65.2%.

^b^Estimated from parametric Cox models. Multivariate model was adjusted for age, center, year of screening exam, smoking status, alcohol intake, physical activity, education level, total calorie intake, family history of diabetes and BMI at baseline.
